# Stop-bang questionnaire for screening obstructive sleep apnea syndrome among hypertensive patients in Kenya

**DOI:** 10.1186/s12890-023-02616-z

**Published:** 2023-09-01

**Authors:** Salim Abdallah Salim, Jasmit Shah, Jumaa Bwika, Sayed K. Ali

**Affiliations:** 1 Department of Medicine, Aga Khan University, P.o box 30270-00100, Nairobi, Kenya; 2 Brain and Mind Institute, Aga Khan University, Nairobi, Kenya

**Keywords:** OSA, Hypertension, Sub-saharan Africa, Sleep disorder, Screening, STOP-Bang

## Abstract

**Introduction:**

Obstructive sleep apnea (OSA) is a type of breathing problem during sleep caused by the blockage of the upper airway, which can cause cessation of airflow. There is limited research on the prevalence of OSA in hypertensive patients in sub-Saharan Africa (SSA). The study aimed to describe the prevalence and clinical characteristics of OSA among hypertensive patients at a tertiary hospital in Nairobi, Kenya.

**Methods:**

This cross-sectional study was conducted at the Aga Khan University Hospital in Nairobi, Kenya. Two hundred and fifty-one hypertensive patients were screened for OSA risk using the STOP-Bang questionnaire (SBQ). Patients with a SBQ score of ≥ 4 were categorized as high risk for OSA. Descriptive statistics were employed to describe both categorical and continuous variables and binary logistic regression to assess factors associated with the high risk of OSA.

**Results:**

The study reported that 78.5% of the participants had high-risk OSA. The median age and body mass index (BMI) were 57.0 years (IQR: 50.0–64.0) and 28.3 kg/m^2^, respectively. Age, neck circumference, gender, and BMI were significantly higher in the high-risk OSA group as compared to the low-risk group.

**Conclusion:**

The study highlights the importance of screening hypertensive patients for OSA using the SBQ in clinical settings, particularly in low-and middle-income countries (LMICs). Healthcare providers can use patient characteristics such as age, gender, neck circumference, and BMI to identify those at greater risk of developing OSA. Further research could focus on developing effective OSA prevention and treatment interventions in hypertensive patients.

## Introduction

Obstructive sleep apnea (OSA) is a prevalent sleep disorder that disrupts normal breathing patterns during sleep, leading to a lack of oxygen and frequent interruptions in sleep cycles. It is characterized by symptoms such as snoring, excessive daytime sleepiness, fatigue, and recurrent upper airway blockages during sleep. OSA has emerged as a significant health concern due to its detrimental effects on multiple physiological systems. The repetitive episodes of hypoxia and sleep fragmentation associated with OSA contribute to various comorbidities, including cardiovascular diseases, cognitive impairments, and impaired daytime functioning [1]. Globally, the estimated overall risk of OSA in the adult population by sleep study ranges from 7.8–77.2% [2] and varies by region [3]. A retrospective hospital-based study among young hypertensive patients in Thailand found a prevalence of 88.9% [4]. In sub-Saharan Africa (SSA), studies showed varied prevalence in different populations ranging from 3% in pregnant South African women, 18% in Nigerian hospital workers, 73% in medical outpatients in Nigeria [5, 6], 24–28% in commercial drivers [7], 37.2% among the general population in Kenya [2], 8% in non-diabetic Kenyans [8], and 37% in Kenyan commercial drivers [9]. However, there was no published data on the prevalence of OSA among hypertensive patients in African countries.

OSA, has been observed to occur in a substantial number of individuals diagnosed with hypertension, with prevalence estimates ranging from 30 to 50% [8]. However, these figures might not accurately represent the true prevalence of OSA in hypertensive patients, mainly due to the under recognition and underdiagnosis of this condition [8]. In a study conducted in Thailand, where a sleep study was employed, it was found that a remarkable 85.7% of hypertensive patients diagnosed in Jinchai region exhibited symptoms of OSA [9]. Furthermore, another investigation conducted in Thailand focused on young participants aged 18–35 with hypertension, and the results revealed that OSA emerged as the primary underlying cause of their elevated blood pressure [10].

According to Robert et al. [10], the prevalence of treatment-resistant hypertension globally is 12-15% using population-based studies and 15-18% using clinic-based reports. Treatment-resistant hypertension (RHTN) is defined as high blood pressure levels that persist despite using three or more medications for hypertension, including a diuretic administered at the maximum dose possible or as the use of at least four antihypertensive medications to achieve the target blood pressure levels [10]. In RHTN patients, the prevalence of severe OSA was found to be 64% [11]. OSA is also common among patients with RfHTN, ranging from 60–90% [12, 13]. In a multicenter study, Martinez-Garcia et al. found that patients with RfHTN were twice as likely to have severe OSA (OR:1.9, 95%CI: 1.02–3.8) than those without RfHTN. The same study estimated the prevalences as 95.2% for moderate and 64.3% for severe OSA in the RfHTN group and 81.8% and 48.6% in the RHTN group, respectively [12]. Refractory hypertension (RfHTN) is described as high blood pressure that cannot be regulated even with the use of at least five medications for hypertension, including a thiazide-like diuretic that has a long-lasting effect and a mineralocorticoid receptor antagonist at the highest dose that can be tolerated [14].

OSA is common in men, with a range of 6–20%, compared to women, who have a range of 6–9% [15]. Several factors have been put forward, postulating the differences in OSA incidence between genders. In general, women tend to exhibit symptoms of OSA that are not directly related to the condition, such as tiredness, headaches, anxiety, depression, and difficulty falling asleep. On the other hand, men tend to display more distinct OSA symptoms like loud snoring, sudden gasping, snorting, and periods of interrupted breathing. Other postulated reasons explaining the higher reporting of OSA among males include increased upper airway collapsibility, increased fluid shifts from the legs to the neck while lying down, and testosterone, which is predisposed by increasing the respiratory response to hypoxia and hypercarbia enhancing respiratory instability [16, 17].

Obesity contributes to the development of OSA, and the progression varies between elderly and younger populations. The prevalence of OSA in normal-weight adults is half that in obese or severely obese patients [18]. Being obese/overweight has been demonstrated to have an increasing effect on OSA, according to a study conducted in Kinshasa (OR:1.94, 95%CI: 1.13–3.78). According to a study, weight gain was found to have a notable influence on the advancement of OSA from mild to severe. In contrast, weight loss was associated with an improvement in the severity of OSA [11, 19, 20]. Neck circumference correlates with severe OSA and is an essential predictor of OSA, as demonstrated in previous studies [20, 21]. A neck circumference of 35.5 cm (17 inches) or greater in men and 32 cm (16 inches) or greater in women is considered a risk factor for severe OSA [22]. Monique et al. found an association between neck circumference and OSA, with men having larger neck circumferences than women [23, 24].

Snoring is the most common, and most looked out for symptoms when screening for OSA. However, snoring is not a symptom specific to OSA, despite being a significant symptom among other risk factors in OSA patients [25]. Better diagnostic accuracy for snoring is witnessed in apnea which is only sometimes present. The most reliable way to diagnose OSA is through a comprehensive overnight polysomnogram (PSG) test conducted during a sleep study. The procedure is monitored all night or partially in the night and is quantified through an apnea-hypopnea index (AHI) that monitors events of apneas and hypopneas per hour [26]. The assessment categorizes the severity of OSA from mild to severe, with mild being defined as AHI of 5 or greater but less than 15 and severe being an AHI of 30 or greater [27]. Despite its importance, the test is not covered by private and national hospital insurance fund medical insurance, thus making it not feasible in the low-and-middle income countries (LMICs).

Several models and screening tools that are easy-to-use and efficient, including SBQ has been developed to screen for OSA. The SBQ assesses multiple risk factors associated with OSA, such as snoring, daytime fatigue, witnessed breathing problems during sleep, high blood pressure, age, body mass index (BMI), neck circumference, and gender. Solecka et al. reported, the SBQ has a sensitivity of 81.6% and a specificity of 75.0% [28] whereas Chiu et al. reported that the SBQ is more sensitive than the BQ and Epworth Sleepiness Scale (ESS) in identifying the severity of OSA from mild to severe. The SBQ emerged as a competent screening and early diagnosis tool for sleep specialists in low-resource settings, especially where PSG is unavailable [29]. To our knowledge, no previous studies have used SBQ to screen the risk of OSA among hypertensive patients in Kenya. This study seek to bridge the gap by investigating the prevalence of OSA and clinical characteristics among in hypertensive attending private tertiary clinics in Kenya using SBQ.

## Methods

### Study design, setting and population

This prospective cross-sectional study was conducted at the Aga Khan University Hospital, Nairobi (AKUHN), between March 2022 and November 2022. AKUHN is a tertiary facility that serves as a postgraduate training institute and a referral center for sub-Saharan Africa. The hospital, which is a component of the global Aga Khan Development Network (AKDN), serves patients with diverse social and demographic characteristics, with most of them accessing care and treatment through private medical health insurance, unlike in the public health sector where national hospital insurance fund (NHIF) medical covers are used. The site was selected because it serves a broad range of internal medicine clinics attending many hypertensive patients. Adults (aged ≥ 18 years) and English-speaking hypertensive patients receiving care and treatment services in the outpatient medical clinics of the hospital were included. Persons with existing/ congenital laryngeal-facial deformities such as retrognathia, macroglossia, maxillary or mandibular hypoplasia, and crowded oropharynx were excluded. A total of 251 participants were included in the study.

### Measurements

Demographic and clinical data such as age, gender, cardiovascular disease, and diabetes mellitus were obtained using a structured questionnaire, and SBQ was administered face-to-face to each study participant as the screening tool for OSA. The questionnaires were validated by the experts working in this area. They were piloted with a set of patients to ensure the constructs were relevant to the intended audience before the start of the actual data collection. The SBQ is a closed-ended questionnaire with eight questions with Yes/No responses and a total score between 0 and 8 points. A score of 1 was given to any question, answering “yes,“ and scores 0 otherwise. Each question that met the criteria of age ≥ 50 years, neck circumference ≥ 40 cm (16 inches), and BMI ≥ 35 kg/m2 was assigned a score of 1. Based on the total score, participants were classified as low-risk (score: 0–3) and high-risk (score: ≥ 4). The explanatory variables included age in years, gender, body mass index, diabetes status, neck circumference, and the likelihood of resistance. Data on transthoracic echo ultrasounds were retrieved from the patients’ files; these results were recorded after the cardiologist’s validation.

Data were collected from Monday to Friday, starting from 9 a.m. to 5 p.m. These are the operating days and hours of the clinics. The number collected daily varied depending on the clinical bookings; the number attended to, and the number of consented patients.

### Statistical analysis

Summary statistics were presented as frequencies and percentages for categorical data and median and interquartile ranges (IQR) for continuous data. The prevalence of high OSA was calculated and reported along with their 95% confidence intervals (CI). Comparison of the two groups (low risk vs. high risk) was determined using Fisher’s exact test or Chi-squared for categorical data and Wilcoxon signed rank sum test for continuous variables. Binary logistic regression was fitted to assess factors associated with the high risk OSA. All significant variables at univariate level and those that are clinically important in explaining the risk of OSA (diabetes and likelihood of resistance) were included in the multivariable model. A p-value of less than 0.05 was considered statistically significant and all analysis was performed using SPSS (IBM Version 23).

## Results

This study analysis involved a total of 251 participants of whom 51.0% (n = 128) were females. The median age of the participants was 57.0 years (IQR: 50.0–64.0). The median neck circumference and BMI were 15.5 inches (IQR: 14.5–16.5) and 28.3 kg/m${\text{}}^{2}$ (IQR: 25.7–31.8) respectively. Of the participants enrolled, 20.3% (n = 51) had clinically diagnosed diabetes mellitus, 5.6% (n = 14) had cardiovascular diseases and 19.5% (n = 49) had resistant hypertension (Table 1).

**Table 1:** Demographics and clinical characteristics of the study participants

Variable	N = 251
**Age (years)**	57.0 (50.0–64.0)
**Gender**	
Male	123 (49.0%)
Female	128 (51.0%)
**Weight (kg)**	78.2 (71.3–87.3)
**Height (m)**	1.7 (1.6–1.7)
**BMI (kg/m** ^**2**^ **)**	28.3 (25.7–31.8)
**Clinically diagnosed diabetes**	51 (20.3%)
**Cardiovascular disease**	14 (5.6%)
**Neck circumference (inches)**	15.5 (14.5–16.5)
**Resistant hypertension**	49 (19.5%)

Data presented as median (IQR) or n(%); BMI: Body Mass Index; IQR: Interquartile range

The responses of the SBQ among the study participants are shown in Table 2. All subjects had high blood pressure. Over three-quarters, 76.1% (n = 191) of the study participants were aged > 50 years, 43.5% (n = 108) each reported often feeling tired, fatigued, or sleepy during the daytime, and had a neck circumference > 40 cm. The prevalence of high-risk OSA was 78.5% (95%CI: 72.9-83.4%).

**Table 2:** Findings of the SBQ among the study participants

Variable		N = 251
**Do you SNORE loudly (Loud enough to be heard through closed doors)?**	Yes	89 (35.5%)
**Do you often feel TIRED, fatigued, or sleepy during daytime?**	Yes	108 (43.5%)
**Has anyone OBSERVED you stop breathing during your sleep?**	Yes	21 (8.4%)
**Do you have or are you being treated for High Blood Pressure?**	Yes	251 (100.0%)
**BMI more than 35 kg/m** ^**2**^ **?**	Yes	30 (12.0%)
**Age over 50 years**	Yes	191 (76.1%)
**Neck circumference > 40 cm (16 inches)? n (%)**	Yes	108 (43.2%)
**Gender: Male?**	Yes	123 (49.0%)
**STOP-Bang criteria**		
**STOP-Bang Score**		4.0 (3.0–5.0)
**Low Risk**		54 (21.5%)
**High Risk**		197 (78.5%)

Data presented as median (IQR) or n (%); BMI: Body Mass Index; IQR: Interquartile range

**Fig. 1:**
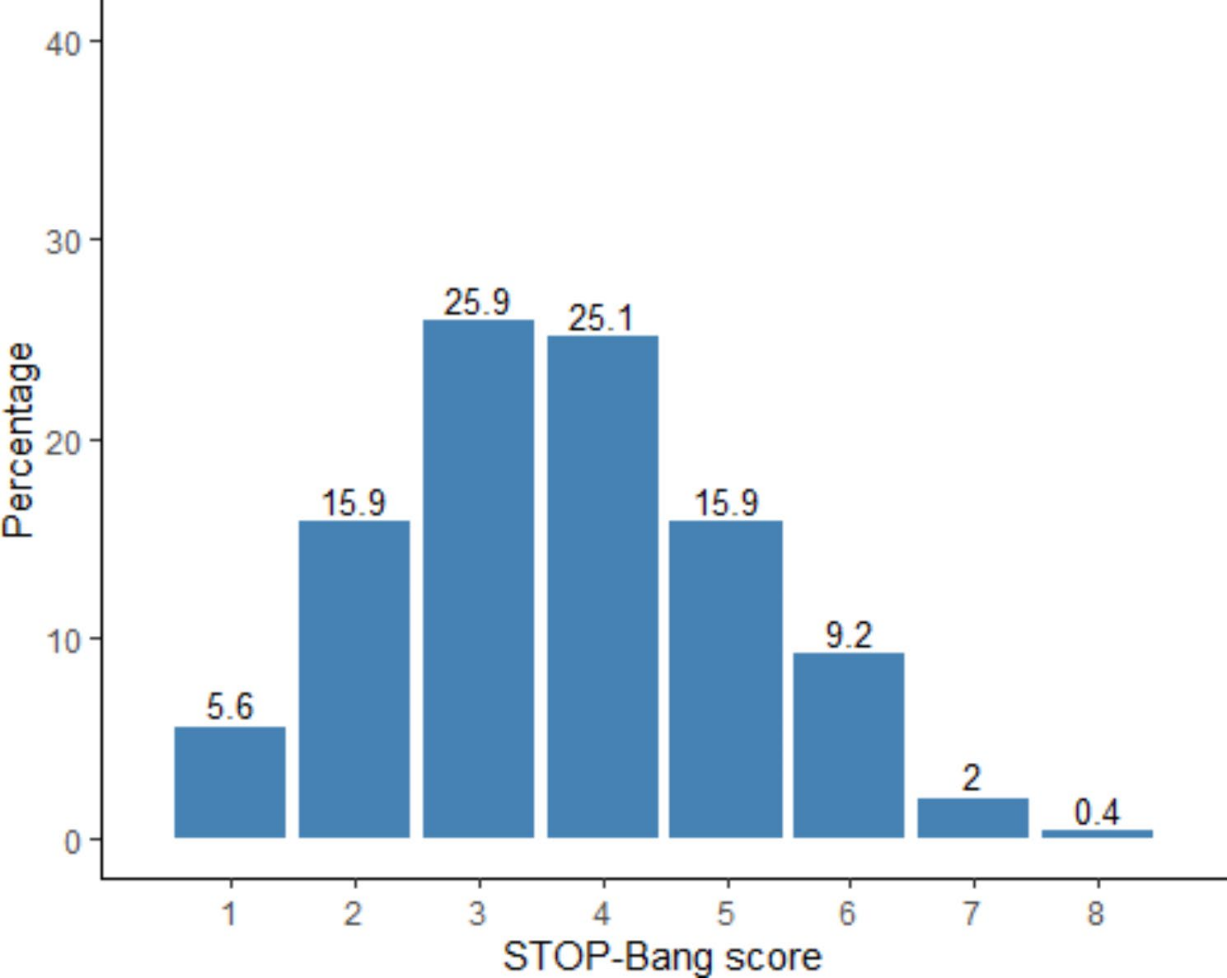
Distribution of participants based on the STOP-Bang scores in 251 hypertensive patients. There were 14 with a score of 1, 40 people with a score of 2,65 people with a score of 3,63 people with a score of 4,40 people with a score 5,23 people with a score of 6, 5 people with a score of 7, and 1 person with a score of 8

The distribution of each SBQ score is shown in Fig. 1. None of the participants had zero scores, 25.9% of the participants had a score of three, followed by a score of four at 25.1%. The median SBQ was determined to be 4.0 (IQR: 3.0–5.0).

### Factors associated with high risk OSA

The proportion of males in the high-risk group was significantly higher compared to the low-risk group (61.4% (n = 121) vs. 3.7% (n = 2); p < 0.001).Similarly, the median age was higher in the high-risk group compared to the low-risk group (58.0 years vs. 49.0 years; p < 0.001). The two groups had a statistically significant difference in median weight (p < 0.001). The high-risk group had significantly high median neck circumferences than the low-risk group (16.0 inches vs. 14.4 inches; p < 0.001). Neither diabetes, resistant hypertension, nor cardiovascular disease were associated with the high-risk OSA (Table 3).

**Table 3:** Stratification of demographic and clinical characteristics between low and high-risk OSA

	OSA		
Variable	low risk, N = 54	high risk, N = 197	p-value
**Age (years)**	49.0 (43.2–56.0)	58.0 (53.0–65.0)	< 0.001
**Gender**			< 0.001
Male	2 (3.7%)	121 (61.4%)	
Female	52 (96.3%)	76 (38.6%)	
**Weight (kg)**	75.3 (68.4–79.2)	80.0 (72.5–90.0)	< 0.001
**Height (m)**	1.6 (1.6–1.7)	1.7 (1.6–1.7)	< 0.001
**BMI (kg/m** ^**2**^ **)**	28.4 (25.5–30.4)	28.2 (25.7–32.1)	0.37
**Clinically diagnosed diabetes**	8 (14.8%)	43 (21.8%)	0.26
**Cardiovascular disease**	1 (1.95)	13 (6.6%)	0.31
**Resistant hypertension**	23 (24.5%)	26 (16.6%)	0.13
**Neck circumference (inches)**	14.4 (14.0–15.0)	16.0 (15.0–17.0)	< 0.001

Data presented as median (IQR) or n(%); BMI: Body Mass Index; IQR: Interquartile range

In the multivariable analysis, all variables except clinically diagnosed diabetes and likelihood of resistance demonstrated significantly associated with high-risk OSA. The odds of having high-risk OSA increased by 7% with each unit increase in participants’ age (AOR: 1.07; 95% CI: 1.04–1.11). Being male was associated with over a four-fold increase in the risk of high-risk OSA (AOR: 4.14; 95% CI: 1.87–9.37). Similarly, a unit increase in BMI was associated with a 12% higher risk of high-risk OSA (AOR: 1.12; 95% CI: 1.03–1.23). Lastly, an increase in neck circumference was associated with more than a two-fold increase in the risk of high-risk OSA (AOR: 2.38; 95% CI: 1.74–3.38) (Table 4).

**Table 4:** Multivariable logistic regression of factors associated with high risk OSA among hypertension participants

		univariable		multivariable	
Variables		OR (95%CI)	p-value	AOR (95%CI)	p-value
**Age (years)**		1.05 (1.03–1.08)	< 0.001	1.07 (1.04–1.11)	< 0.001
**Gender**	Female	Reference			
	Male	5.17 (3.04–8.97)	< 0.001	4.13 (1.87–9.37)	0.001
**BMI**		1.10 (1.04–1.17)	0.002	1.12 (1.03–1.23)	0.011
**Diabetes**	No	Reference			
	Yes	1.68 (0.90–3.21)	0.106	0.91 (0.38–2.18)	0.832
**Neck circumference (inches)**		2.81 (2.15–3.79)	< 0.001	2.38 (1.74–3.38)	< 0.001
**Likelihood of resistance**,	No	Reference			
	Yes	1.02 (0.55–1.92)	0.941	1.17 (0.49–2.86)	0.722

### OSA and clinical characteristics findings

The description of 2D-echo findings among the participants is shown in Table 5. Of the enrolled participants, 23.3% (n = 58) had a 2D-echo ultrasound performed at the institution over the last year before the study. Left atrial enlargement was found in 46.6% (n = 27) and 43.1% (n = 25) had diastolic dysfunction. Only 15.5% (n = 9) of the participants had a reduced ejection fraction, 8.6% (n = 5) had mid-reduced, and 75.9% (n = 44) had normal ejection fraction. The median Right ventricular systolic pressure (RVSP) was 29.1 mmHg (IQR: 23.9–33.7).

**Table 5:** 2D-ECho findings among participants that had transthoracic echocardiography performed at AKU in 2021

Variable	N = 251
**2D-echo ultrasound done**	58 (23.3%)
**Left atrial enlargement**	27 (46.6%)
**Ejection Fraction**	
Reduced	9 (15.5%)
Mid-reduced	5 (8.6%)
Normal	44 (75.9%)
**Diastolic dysfunction**	25 (43.1%)
**RVSP (mmHg)**	29.1 (23.9–33.7)

Data presented as median (IQR) or n(%); BMI: Body Mass Index; IQR: Interquartile range; RVSP: Right ventricular systolic pressure

Table 6 summarizes the 2D-echo ultrasound findings stratified by low and high-risk OSA groups. There was no statistically significant difference in ultrasound findings and the two groups (low-risk and high-risk groups). All patients with low-risk OSA had a normal ejection fraction, whereas, among the high-risk group, 68.2%(n = 30) had normal ejection fraction. Patients with left atrial enlargement were more among the high-risk group than in the low-risk group (46.9% (n = 23) vs. 28.6%(n = 4)).

**Table 6:** The stratifications of transthoracic Echocardiography findings and risk of OSA groups

Variable	Low-risk, N = 54	high-risk, N = 197	p-value^1^
**2D-echo ultrasound**	14 (25.9%)	44 (22.6)	0.61
**Left atrial enlargement**	4 (28.6%)	23 (46.9)	0.22
**Ejection Fraction**			0.06
Reduced	0 (0.0%)	9 (20.5%)	
Mid-reduced	0 (0.0%)	5 (11.4%)	
Normal	14 (100.0%)	30 (68.2%)	
**Diastolic dysfunction**	5 (38.5)	20 (41.7)	0.83
**RVSP (mmHg)**	29.4(24.3–39.1)	28.9 (24.1–33.7)	0.89

Data presented as median (IQR) or n(%); BMI: Body Mass Index; IQR: Interquartile range

## Discussion

Intermittent cyclic hypoxia and disruption of sleep patterns characterize OSA, a widespread syndrome with population health burdens over the years. The syndrome is underdiagnosed and undertreated worldwide, even among patients with underlying conditions such as hypertension, even though it can initiate or alter the development of other long-term illnesses, such as systemic arterial hypertension [2]. The case is not different among participants with hypertension in this study setting. The research employed the SBQ to gauge the likelihood of OSA in individuals with hypertension and found a high prevalence of high risk of OSA at 78.5%. Notably, two studies in the Thai region examining young and elderly hypertensive participants also reported a high prevalence of OSA using sleep studies of 88.98% and 85%, respectively [4, 30].

Another study conducted in Nigeria using the ESS as a screening tool found a high prevalence of OSA at 50% among hypertensive participants [31]. Similarly, a cross-sectional study in Ethiopia reported a prevalence of 63.3% of OSA in the hypertensive group using SBQ [32]. These studies suggest a consistently high prevalence of OSA in hypertensive individuals across different regions, albeit with different screening tools employed. The study showed significant differences in age, gender, weight, height, and neck circumference between the low and high-risk groups.

OSA was more prevalent among males than in females. The finding agrees with other studies demonstrating a male predominance of OSA [16, 17]. Men tend to indicate symptoms of OSA, such as snoring, while women are likelier to report symptoms unrelated to OSA. In addition, the role of testosterone hormones has been reported to enhance respiratory instability [16, 17].

OSA increases with age due to changes in organ functions that occur with age and affect sleep neurophysiology [33]. Specifically, old age is often accompanied by muscular and neurological loss of muscle tone of the upper airway with enhanced collapsibility, thus increasing the risk for OSA in adult patients [33]. The median age was higher in the high-risk group than in the low-risk.

The study found a larger neck circumference among the high-risk group than the low-risk group. The finding was similar to other studies that demonstrated that a larger neck circumference positively correlated with the risk of developing OSA [16, 23, 24, 34]. People with a larger circumference around their necks may have extra fat stored around the upper airway, which can restrict breathing and cause snoring when lying on their back, increasing the likelihood of developing OSA.

There was a notable difference in people’s body weights classified as high-risk and low-risk for OSA. In both cases, the median weight (kg) among the low-risk group was lower than the high-risk group. A study conducted in Kinshasa found a greater chance of developing OSA for individuals who were overweight or obese [35]. A study conducted in Thailand among young hypertensive patients showed that the severity of OSA increased with the BMI [4]. Excess weight, especially pharyngeal fat, and increased abdominal girth compress the neck, diminishing the airflow, and making the upper airway more likely to collapse during sleep, therefore predisposing to the development of OSA [36, 37] .

There was no association between cardiovascular disease and increased risk of OSA. However, the prevalence was higher in the high-risk groups than in low-risk groups. The echocardiography showed a higher proportion of individuals in the high-risk OSA group having left atrial enlargement, reduced ejection fraction, and diastolic dysfunction than the low-risk group. However, the difference was not statistically significant. Nonetheless, the median RVSP was not significantly different between the two groups. These results contradict previous studies that have demonstrated an increased incidence of chronic heart failure, as indicated by left atrial enlargement, high systolic pulmonary arterial pressure, and diastolic dysfunction in patients with OSA [38–42]. The dissimilarity in this result between the present study and studies conducted in other settings may be attributable to the limited number of participants who underwent a 2D-echo ultrasound in this study, resulting in insufficient statistical power to detect a significant difference.

The current study defined resistant hypertension as using four or more antihypertensive drugs by the patient for blood pressure control. Contrary to Wu et al. [43] findings, there was no association between resistant hypertension and the risk of OSA. This could be attributed to incomplete information on the number of antihypertensive drugs participants used on the clinical visit progress notes.

### Strength and limitation

The study utilized screening tools instead of polysomnography to assess OSA, potentially introducing limitations to its precision. Nevertheless, these screening tools have undergone validation and can function as reasonable proxy indicators for OSA. The study was conducted within the confines of a private tertiary clinic, thereby constraining the extent to which the outcomes can be generalized. This suggests that our findings might only partially represent the broader population or encompass the array of diverse healthcare settings.

Moreover, given the study’s setting in a private tertiary clinic, the results may only partially apply to healthcare environments with fewer resources and differing standards of care. The conclusions drawn regarding resistant hypertension hinged on patients’ recollections regarding their antihypertensive medications, coupled with a charted inventory of these medications in the patients’ clinic review notes, as recorded by the attending physician. This approach opens the door to potential recall bias and incomplete records, which could have influenced the accuracy of the results.

Furthermore, the participants’ findings on the transthoracic echo were contingent upon whether they had undergone the echocardiogram at AKUHN and whether this had occurred within the past year. This temporal restriction impacted the comprehensiveness of the data gathered concerning cardiovascular-related changes. The study ultimately identified no discernible correlation between diabetes, cardiovascular disease, resistant hypertension, and the risk of OSA. This could be attributed to the relatively modest size of the participant pool afflicted with these conditions, alongside the abovementioned limitations.

In order to gain a more definitive understanding, it is recommended that future research be conducted with a more substantial cohort of patients. Such an approach would aid in elucidating the present findings and addressing the intricacies introduced by the limitations outlined above.

## Conclusion

In conclusion, the study found a high prevalence of high-risk OSA among participants. Age, BMI, neck circumference, and gender were associated with the high risk of OSA. The study recommends conducting further studies to include other private tertiary clinics not related to Aga Khan University and public hospitals to validate the findings of this study. Another recommendation is to use SBQ for OSA screening among all hypertensive patients in the appropriate clinical settings. Additionally, the study recommends the availability and affordability of sleep studies in our settings.

## Data Availability

All data used in this study can be obtained through written request to the corresponding author.

## Abbreviations

AHI: Apnea-Hypopnea Index
AKUHN: Aga Khan University Hospital, Nairobi
BMI: Body Mass Index
CI: Confidence Interval
CPAP: Continuous Positive Airway Pressure
ESS: Epworth Sleepiness Scale
ISERC: Institutional Scientific and Ethics Research Committee
IQR: Interquartile Range
LMIC: Low- and middle-income countries
NACOSTI: National Commission for Science, Technology, and Innovation
OR: Odds Ratio
OSA: Obstructive Sleep Apnea
PSG: Polysomnography
RfHTN: Refractory hypertension
RHTN: Resistant Hypertension
RVSP: Right ventricular systolic pressure
SSA: Sub-Saharan Africa
SBQ: STOP-Bang Questionnaire
